# COVID-19 Vaccines and the Virtues

**DOI:** 10.1093/phe/phac027

**Published:** 2022-11-14

**Authors:** Konrad v Boyneburgk, Francesca Bellazzi

**Affiliations:** Department of Philosophy, King’s College London, London, UK; Department of Philosophy, University of Bristol, Bristol, UK

## Abstract

From a moral point of view, what arguments are there for and against seeking COVID-19 vaccination? Can it be morally permissible to require (parts of) a population to receive a vaccine? The present paper adopts a perspective of virtue ethics and argues both that it is morally right for an individual virtuous moral agent to seek COVID-19 vaccination and for a virtuous ruler to impose mandatory vaccinations on her population.

We begin by first presenting virtue ethics and the current vaccine controversy. Second, we examine whether a virtuous individual should get vaccinated. Third, we consider whether, from a moral point of view, it is right for a ruler to impose mandatory vaccinations on her citizens. Fourth, we answer some objections to our argument. Finally, we conclude that virtue ethical considerations warrant both the individual choice of getting vaccinated and mandatory vaccinations against COVID-19.

Several vaccines against SARS-CoV-2, the virus that causes COVID-19, are available since late 2020. Suspicion against them is still widespread over social networks and some media. Governments have responded with information and advertising campaigns and, more recently, by making vaccination mandatory for some groups of the population such as health workers or by introducing forms of vaccination passports. In some countries, such as Italy, vaccination is mandatory for those above 50 years of age since February 2022 and similar measures have been discussed worldwide.

From a moral point of view, what arguments are there for and against seeking COVID-19 vaccination? Can it be morally permissible to require (parts of) a population to receive a vaccine? The present paper adopts a perspective of virtue ethics and argues both that it is morally right for an individual virtuous moral agent to seek COVID-19 vaccination and for a virtuous ruler to impose mandatory vaccinations on her population.

We first present virtue ethics and the current vaccine controversy. Second, we examine whether a virtuous individual should get vaccinated. Third, we consider whether, from a moral point of view, it is right for a ruler to impose mandatory vaccinations on her citizens. Fourth, we answer some objections to our argument. Finally, we conclude that considerations of virtue support both individual vaccinations and vaccination mandates against COVID-19.

## The Vaccine Controversy and Virtue Ethics

The current SARS-CoV-2 (COVID-19) pandemic is one of the biggest challenges we have faced in recent years. At the time of writing, *The Economist* estimates that COVID-19’s actual death toll is 22.9 million lives (The Economist, 2021). A recent report published in *The Lancet* has discovered that more than 7,653,000 children in the world have lost one or both of their parents (Unwin *et al.*, 2022). It seems evident that it is a world-wide priority to exit this situation as soon as possible. This is possible via a comprehensive vaccination campaign with safe and effective vaccines that can contain the virus.

Thanks to the work of many laboratories and scientists, different COVID-19 vaccines with sufficiently corroborated efficacy are available. Additionally, at least in the Western world, it is possible to organize relatively efficient vaccination campaigns. However, scepticism and disinformation concerning vaccines is widespread on social media and among some groups. This has generated resistance to vaccination campaigns and produced a delay in the uptake of vaccines in some countries. As a response, governments provided different forms of benefits to those that receive vaccinations (Politi and Wells, 2021; Savulescu, 2021) and started implementing mandatory vaccinations for some subsets of their populations. Still, whether to seek vaccination against SARS-CoV-2 remains an individual choice in most parts of the world.

Whereas the situation in each country would warrant its own analysis that takes economic and legal aspects into account, we can ask whether, from a moral point of view, it is right for an individual to get vaccinated and for a ruler to impose mandatory vaccinations. To explore these questions, we use the moral framework of virtue ethics, an approach with innovative contributions to ethical issues during the pandemic. Thus far, this approach has not been applied to arguments concerning vaccinations against COVID-19 in the literature. In contrast with other moral frameworks, such as deontology or utilitarianism, virtue ethics regards as morally fundamental the character and consequent behaviour of a morally virtuous person, where the virtues are excellent character traits that dispose their possessor to feel and act in certain ways in a given situation (Bellazzi and v. Boyneburgk 2020: 4). Virtues are key in considerations of value and moral behaviour. According to virtue ethics, the right action to perform is the one that results from the application of the virtues relevant to the circumstances at hand (ibid.).^1^ In addition, central to virtue ethics are a focus on practical wisdom and the search of happiness. The former is the capacity to correctly practice virtues and to exercise control over one’s life (Hursthouse and Pettigrove, 2018). Concerning the latter, we follow an eudaimonist approach to virtue ethics inspired by Aristotle according to which the goal or purpose of moral behaviour is to reach one’s happiness or flourishing as well as to collaborate at the community level such that this stage can be achieved by others. Happiness of the community or the common ‘good of the city is apparently a greater and more complete good to acquire and preserve’ (Aristotle and Irwin 1999: I.2, 1094b8f.).

Aristotle implicitly offers three reasons as to why the virtues are part of happiness: his inclusive understanding of eudaimonia, the human function argument and his insight that the human being is a political animal. First, eudaimonia, translated either as happiness or ‘living well’, is understood as ‘activities [in] accord with complete virtue, with an adequate supply of external goods […] for a complete life’ (ibid. I.10, 1101a15-17). Aristotle regards happiness as the highest good achievable by human beings. As such, it is pursued for its own sake or ‘complete’ (ibid. I.7, 1097a27-34). It includes other goods that are both chosen for themselves but additionally for the sake of happiness. If a happy life is to include all human goods, an otherwise ideal life lacking in moral virtue will be improved in this regard. Second, Aristotle argues that human beings have a specific function (‘ergon’), consisting in rational activity (ibid. 1098a7). In order to perform that function well, the human being needs virtues (1098a12), the best rational government over her life. Despite having faced intense scrutiny, the function argument can be defended (see e.g. Whiting, 1988). Third, happiness is self-sufficient in that it takes our social nature into account (Aristotle and Irwin, 1999: I.7, 1097b10-12). We wish to treat well both those closest to us and our fellow human beings and hope to be reciprocated in our virtuous behaviour towards them. Moral relationships with fellow human beings are integral part of the happy life. In addition, the present paper suggests the following indirect link between the virtues and happiness: since the virtuous person will seek vaccination against COVID-19, her being virtuous leads to her health (at least in the sense of lowering the likelihood of falling seriously ill from COVID-19). Health is a precondition or necessary component of eudaimonia. Aristotle criticises those who claim we can be happy while falling ‘into terrible misfortunes’ (ibid. VII.13, 1153b20). By analogy, we cannot be happy if debilitated by COVID-19. The advantages of the virtue ethical approach include its focus on the actions of each individual and her flourishing as well as its flexibility in allowing for different behaviours to be relevant in different contexts.

Whether an action such as getting vaccinated is virtuous depends on the given circumstances, including the properties of the vaccines. The currently available evidence suggests the following, especially about Pfizer-BioNTech’s Comirnaty vaccine (cf. Brennan, 2018: 37). This vaccine (i) is highly effective in offering protection against severe illness, hospitalisation and death (Buchan *et al.*, 2022; Collie *et al.*, 2021;Rosenberg *et al*., 2022,)—there may be some protection against symptomatic disease after a booster (Andrews *et al.*, 2022; Garcia-Beltran *et al.*, 2021; Khoury *et al.*, 2021), (ii) possesses few relatively mild side effects while cases of severe side effects including death remain rare (Menni *et al.*, 2021; Xu, *et al.*, 2021). The vaccine (iii) has been produced without inflicting unjust harm on anyone. Finally, (iv) access to vaccines is widely available, at least in Western countries. At the same time, the existing evidence for these properties, while requiring some research, is persuasive and readily available. While (i) will be discussed in the next section, let us consider (ii), (iii) and (iv) in detail.

(ii) According to recent research, the available vaccines possess mild side effects in the overwhelming majority of cases. The European Medicines Agency registered the administration of 665,000,000 doses of Pfizer-BioNTech’s Comirnaty vaccine and a total of 919,242 ‘cases of suspected side effects’, a proportion of 0.1382 per cent (European Medicines Agency, 2022). The US Centres for Disease Control and Prevention registered the administration of 624,000,000 doses of COVID-19 vaccines and 16,803 ‘preliminary reports of death’ among their recipients, a proportion of 0.0027 per cent (Centers for Disease Control and Prevention, 2022a). In addition, they found 105.9 cases of myocarditis in males aged 16–17 per one million doses of the Pfizer-BioNTech vaccine, a proportion of 0.0106 per cent. This compares with a rate of ‘as high as 450 per million’ among young males infected with COVID-19 (Singer *et al.*, 2022). Therefore, the risk of developing heart inflammation from COVID-19 is higher than that from the vaccine (Pyle and Huang, 2022). However, a case by case risk-benefit assessment should be performed, especially in the case of children. While the Centers for Disease Control and Prevention recently approved the vaccines for children over the age of five (Centers for Disease Control and Prevention, 2022b), some worries about vaccinating a group of the population generally perceived to be at lower risk from developing severe COVID-19 remain.

(iii) The vaccine has been produced without inflicting unjust harm on anyone. However, members of some religious communities have objected to the use of foetal tissue obtained from abortion in the research of the vaccine. We can address these concerns in three ways. First, while a relation between religious beliefs and moral principles exists, the two do not map onto each other either fully or directly. There might be an action that is virtuous or morally right even if it does not follow from religious beliefs or principles and there might be religious beliefs and principles that differ from morality. Second, one can still accept a given medical device (such as a vaccine or painkiller) as ethical even if one does not approve of the specific contingent way in which the device is researched (or produced). In this case, one could still accept the usage of vaccinations while condemning a particular research that led to these discoveries since the use of foetal tissue was not essential. The foetal tissue is not a necessary feature or constituent element of the vaccines or their application but was solely employed in part of the relevant scientific research. Third, some religious authorities have responded to these concerns by defending the usage of vaccination. The Islamic Indonesian Ulama Council deemed Pfizer-BioNTech’s vaccine ‘Haram-permittable’, acceptable to use because of the urgency of the COVID-19 pandemic (Mardian *et al.*, 2021). The Catholic Church judged these vaccines morally acceptable since, among other factors, the ‘cooperation in evil’ involved in taking them is remote (Congregation for the Doctrine of Faith, 2020).

(iv) Access to vaccines is widely available, at least in Western countries. Where it is not, vaccines should be made available quickly, including via donations. Moreover, an important presupposition to our argument is that vaccines must be available to the agent in order to be able to morally evaluate her vaccination decision. The presence of the moral choice—in this case whether to accept or refuse an *available* vaccine—represents a necessary condition for the application of virtue ethics. No one who cannot get vaccinated on the grounds of being unable to access a vaccine against COVID-19 can be blamed for that.

Our subsequent argument is conditional upon this evidence. Furthermore, we maintain that equal access to vaccination should be guaranteed together with exemption for those with particular medical conditions that prevent them from safely being vaccinated. Whether those with strong natural immunity from previous infection with SARS-COV-2 can be exempted from mandatory vaccination should be carefully debated: On the one hand, they profit from protection comparable to that of the vaccinated (Pugh *et al.*, 2022). On the other hand, some evidence suggests that the protection afforded against symptomatic infection with Omicron by a combination of vaccination and infection may be greater than from infection alone (Altarawneh *et al.*, 2022; Plumb *et al.*, 2022).

## The Virtuous Get Vaccinated

In this section, we explore whether it is virtuous—and thus morally right—to seek vaccination for oneself by outlining some of the virtues that seem to be especially relevant in the current pandemic. Whether the choice of accepting vaccination against COVID-19 is virtuous or not is important not only at the individual level, but also to determine the actions and policy instruments available to a virtuous ruler or legislator.^2^ Specifically, such a ruler cannot mandate actions that would be vicious for her citizens to perform on pain of forfeiting her being virtuous. Thus, showing that accepting a vaccine against COVID-19 is a virtuous (or neutral) choice is a precondition for any vaccination mandate.^3^ We discuss the latter in the next section. Here, we consider the virtues of prudence, generosity, justice and bravery in an individual. These are relevant since they either regard actions with an impact on others (prudence, generosity and justice) or concern difficult or dangerous situations (specifically bravery and prudence).

Prudence, according to Aristotle, is the capacity to carefully and correctly deliberate about what benefits oneself and one’s surrounding community (Aristotle and Irwin, 1999: VI.5–13, 1140a25ff.). He defines it as ‘a state grasping the truth, involving reason, concerned with action about things that are good or bad for a human being’ (ibid., 1140b5ff.). Prudence is the virtue that enables us to take appropriate action concerning our good and that of others in light of the available evidence. In case the latter changes, prudence may indicate different actions. It thus has a dynamic role in reacting to incoming information as the pandemic evolves, especially with consideration of the Omicron and future variants. As long as what we know about COVID-19 and the vaccines distributed as protection against it remains in line with current evidence as presented above, three reasons suggest it is prudent to seek vaccination.

First, in most cases the vaccines afford reliable protection against severe illness and death. Acquiring such protection is advantageous for oneself. Second, the vaccinated are less likely to require hospitalisation, freeing up hospital beds for other use. The availability of this important resource to others benefits the community. Third, some evidence suggests that vaccinated people are less likely to have a high viral charge (Prunas *et al.*, 2022), including those infected with the Omicron variant (Lyngse *et al.*, 2021; Puhach *et al.*, 2022), thus reducing the risk of infecting others with possibly severe forms of the disease (see (i) above). However, there is conflicting evidence indicating that even those who received a booster dose of a vaccine possess viral loads comparable to the unvaccinated (Acharya *et al.*, 2022; Hirotsu *et al.*, 2022; Singanayagam *et al.*, 2022). The study by Puhach *et al*., however, challenges these latter studies that indicate comparable viral loads in vaccinated and unvaccinated alike: They rely on measuring virus RNA levels that offers ‘only a weak proxy for infectiousness’ (p. 2) and correlates with ‘infectious virus shedding’ only poorly (p. 7). Instead, Puhach *et al*. assess infectious viral load by isolating the virus in cell culture and find that individuals who received a booster vaccine had significantly lower viral loads after infection with Omicron than their unvaccinated counterparts. Whatever the final verdict on this issue, the virtue of prudence can help us exercise caution in contexts of uncertainty. Consequently, the prudent person will most probably seek vaccination in light of this mixed evidence.^4^

Aristotle suggests that what we realize prudence commands possesses a binding quality: ‘prudence is prescriptive, since its end is what action we must or must not do’ (Aristotle and Irwin, 1999: VI.10, 1143a9f.). This implies a certain obligation to perform the actions prudence indicates. Once prudence endorses a given action, a strong form of moral motivation follows and agents will feel morally motivated to pursue this action. In this case, if prudence commands vaccination as the moral choice, then the agent will feel morally motivated to accept it.

Having the choice of getting vaccinated may present an opportunity to exercise generosity. This virtue is primarily concerned with sharing one’s wealth with others (ibid. IV.1.18, 1120b5f.) but can be naturally extended to cover any sacrifice one accepts for the benefit of the surrounding persons (Bellazzi and v. Boyneburgk 2020: 5). Whereas for some people the choice of getting vaccinated can only be evaluated in terms of personal benefits gained, for instance immunity or lower risk of serious disease, for others it implies accepting some sacrifices. These can consist in enduring any side effects, accepting the low but existent risk of complications, travelling to a vaccination centre or a general practitioner, waiting for one’s turn etc. These sacrifices relate to the virtue of generosity as long as they are made *in the intention* of thereby decreasing the likelihood of infecting others or of requiring medical resources in short supply. The former aims at making it less likely to become a vector of the virus, the latter at allowing others in need to use the health system, whether because of COVID-19 or for other reasons. The choice of being vaccinated may thus be generous if one accepts sacrifices in order to benefit the community.

One might object that the virtue of bravery runs counter to our account thus far. Is it not courageous to ‘stand up to the virus’, continue to live one’s life in the face of the threat and thereby risk infection? Some rhetoric urging us to ‘live with the virus’ implicitly appeals to growing out of cowardice into courage. However, a brave person, Aristotle underlines, does fear ‘the right things […] at the right time’ (Aristotle and Irwin, 1999: III.7, 1115b17-19).^5^ The truly brave agent shuns genuine threats. Risking infection and spreading this virus is not courageous but rash, as Aristotle calls the person who is excessively confident towards what should be feared (ibid. 1115b29f.), especially since considerable uncertainty besets whether each one of us will fall severely ill with COVID-19 or develop Long COVID, its chronic form. While we may be able to determine the risk of severe illness of certain groups on the grounds of age or pre-existing health conditions, as much is not possible at the individual level. Any safety that the unvaccinated may think they have is only apparent, as they have, *ceteris paribus*, higher chances of becoming severely ill. Thus, meeting COVID-19 ‘unarmed’, so to say, is reckless. As a result, bravery is not an objection against a virtuous person seeking immunization against COVID-19. On the contrary, the brave person is aware that catching this disease may have serious consequences for herself and others and for this reason chooses protection instead of merely ‘living with it’.

Finally, Aristotle refers to the virtue of justice as the capacity not to overreach, i.e. to gain undue profit at the expense of others (ibid. V.2.1130a33). It is concerned with the distribution of divisible goods in a community (ibid., 1130b30ff.). A contemporary interpretation consistent with virtue ethics would supplement the fair distribution of burdens (Savulescu, 2021: 82, but cf. Aristotle and Irwin, 1999: V.5, 1134a2ff.). Accordingly, refusal to obtain vaccination against COVID-19 constitutes unjust behaviour since it draws an unfair profit ‘concerned with […] safety’ (ibid., V.2, 130b32ff.): First, one benefits from the diminished likelihood of catching the virus from the vaccinated as well as availability of scarce health care resources without assuming the burden of getting vaccinated oneself.^6^ Such free-riding is unjust since it reaps illegitimate profit (unless there are relevant health-conditions that prevent one from being vaccinated) and thereby morally wrong in the virtue ethical sense. Second, exposing others to the risk of being infected with a high viral charge of COVID-19 against their will is unjust since it inflicts unwarranted and avoidable harm on them. Third, COVID-19 has a significant impact on the health system, preventing it from working efficiently. Thus, the behaviour of the unvaccinated could result in taking up needed space in hospitals, preventing the correct distribution of health care resources (e.g. operations and screenings). The first two points are weakened in the light of the above mixed evidence on the infectiousness of the vaccinated with breakthrough infection. But as long as it is probable that, when infected, the vaccinated possess a lower viral load than the unvaccinated and thus a diminished probability of infecting others, these points cannot be dismissed. Instead, they merely become probable instead of certain.

In conclusion, a virtuous agent uses prudence, generosity, bravery and justice during the current pandemic and these virtues support the choice of being vaccinated. Given that within the framework of virtue ethics the morally right action is one a virtuous agent would choose, getting vaccinated is morally right. An individual virtuous moral agent should seek COVID-19 vaccination.

## A Virtuous Ruler and Mandatory COVID-19 Vaccinations

Let us turn to a ruler or legislator who faces the decision whether to impose mandatory COVID-19 vaccinations on her population. Would this course of action be virtuous and thus morally right according to the ethical framework here employed? In order to answer this question, we assume that the ruler is endowed with the relevant virtues as well as legally and constitutionally permitted to pursue this course of action. It is a trivial consequence that if imposing mandatory vaccination against COVID-19 is a virtuous choice, then it can be applied by a virtuous ruler. This section argues that imposing mandatory vaccination is a virtuous choice for a ruler.

An application of virtue ethics at the institutional level has been traditionally supported by Aristotle, but also by contemporary philosophers. Swanton, for instance, writes that any social institution should—according to virtue ethics—‘serve the worthwhile ends for which they are (or ought to be) designed’ (Swanton, 2003: 33). It is part of the purpose of a social institution, such as the government, to preserve the health of the population. This includes protecting the vulnerable, the immunocompromised and all those who do not respond to receiving the vaccine with the development of antibodies and the avoidance of Long COVID that is still a possible effect even of Omicron. Consequently, a ruler should apply norms that reach such a good. This is normally achieved by well-designed laws that aim at advancing the common good of happiness. The relevant laws, Aristotle writes, ‘prohibit actions in accord with the vices’ (Aristotle and Irwin, 1999: V.1, 1129b25). The government’s goal of preserving health must be carefully balanced with maintaining individual liberties in a way we shall see below.^7^

If our previous analysis is correct and it is virtuous to accept vaccination against COVID-19, refusing it without good (medical) reason is vicious. This can be seen particularly well when considering that the refusal of getting vaccinated is unjust, as argued in the previous section. While the distinction between legal and moral principles can be retained—the two are not co-extensive—laws should be informed by moral considerations (‘most lawful actions […] are those produced by virtues as a whole; for the law [i.e. correct legislation] prescribes living in accord with each virtue’, ibid. V.2, 1130b23f.).^8^ Specifically, a ruler might implement laws that prohibit the vicious behaviour of shunning the vaccination. These fall under the scope of those that further the common good, that, in the words of Aristotle, ‘aim […] at the common benefit of all’ (ibid. V.1, 1129b16). Moreover, such laws are not only prohibiting vicious behaviour, but also protecting and encouraging the pursuit of the common good. While this is not a necessary feature of every law—since states might pursue different goals—we maintain that a virtuous ruler would implement laws that protect the common good. Good health and access to health care are preconditions of individual happiness, hence part of the common good. They may be promoted by making vaccination against COVID-19 mandatory. While the virtuous ruler’s task is to make her citizens good, Aristotle seems to suggest a certain dose of lawful compulsion for those unimpressed by persuasion (cf. ibid. X,9, 1180a5f.) and advocates ‘corrective treatments and penalties on anyone who disobeys’ (l.9f.).

From a moral point of view, the above considerations on prudence can be extended directly to the institutional level (Bellazzi and v. Boyneburgk, 2020): In light of the available evidence, a virtuous ruler has to take appropriate action to promote the good of her community. The role of prudence is fundamental in this: If the available evidence changes, so does the virtuous response to it. Again, we underline that our arguments are conditional upon the medical evidence as presented above. From the ruler’s perspective, as long as the common good of happiness is threatened by repeated outbreaks of a severe disease such as COVID-19, the policies prescribed by prudence should be followed. Since the virtuous person is the measure of good ethical conduct, her prudent realization to seek vaccination can be required of those who do not share her insight since they lack the relevant virtues (while balancing this goal with others the government may have, cf. Aristotle and Irwin, 1999: VI.8). Mandatory vaccinations increase the proportion of the vaccinated population, thereby preventing hospitals from being overwhelmed and protecting the immunocompromised as well as those parts of society that either cannot receive vaccination or do not respond to it by developing antibodies. The introduction of vaccination passports and certificates to access work or other activities was effective in raising the vaccination rate against COVID-19 (Oliu-Barton *et al.*, 2022), compared with mixed evidence for the impact of financial incentives or nudges (Karaivanov *et al.*, 2022). Specifically, such certificates led to an increment of vaccination both in anticipation and after introduction, with an effect that lasted for more than 40 days (Millis and Rüttenauer, 2022). This is in line with data from the introduction of vaccine mandates against varicella in the USA (Abrevaya and Mulligan, 2011). On a more general level, pointing to a group of people who will remain unvaccinated despite a mandate and claiming that mandatory vaccination is therefore useless cannot be an argument against mandates. As Giubilini (2018: 113) correctly observes, it rather highlights the need for compliance controls and sufficiently severe penalties for remaining unvaccinated. In addition, a high vaccination rate indirectly decreases the risk of the emergence of new dangerous variant strains of the virus by offering it a reduced population, crippling its evolutionary potential (cf. Van Oosterhout *et al.*, 2021: 2013), which in turn benefits the populations of other nations where these variants could spread.

Concerning bravery, the virtuous ruler is implementing a policy that is unpopular at least with a relevant and vocal minority. The consequences can range from threats against her personal safety over intimidation and violent protests to risking her re-election and career as a politician. The virtuous ruler must put threats to her own person into perspective with those posed by the spread of COVID-19 in a partially unvaccinated population.

Regarding justice, a virtuous ruler should address unjust distributions of benefits and burdens that are detrimental to the common good. She may intervene to block unvaccinated free riders from imposing unfair burdens on others (such as otherwise avoidable restrictions of public life or a scarcity of health care resources) and require them to provide their just contribution to the community’s good in order to protect the preconditions of happiness. In the words of Aristotle, ‘just is whatever produces and maintains happiness and its parts for a political community’ (Aristotle and Irwin, 1999: V.1, 1129b18f.). This seems to be consistent with imposing mandatory vaccination when the circumstances are appropriate. Moreover, every single eligible citizen is treated with equal concern by being required to receive a protecting vaccine, thus distributing both the benefit and burden of the vaccination fairly and equally across the population (cf. Giubilini, 2018: 103).

In conclusion, it seems that a virtuous ruler or legislator is right to impose mandatory COVID-19 vaccination via approved laws if this preserves the common good, since this is part of her role and responsibility. Rendering vaccination against COVID-19 mandatory is a virtuous choice.

## Objections

An objection could be marshalled against the imposition of vaccines on supposedly virtuous agents in the population. By making vaccination mandatory, these virtuous moral agents are deprived of the good of freely seeking vaccination themselves and thereby exercising the virtues analysed above. Additionally, Aristotle observes that forced actions cannot be virtuous (cf. Aristotle and Irwin, 1999 III.1, 1110a3f.). The virtuous ruler thus disrespects her virtuous citizens who, following the above arguments, she should assume will pursue vaccination on their own account. However, first, at least in Western countries there has been ample time to seek vaccination of one’s own initiative. Further waiting for the unvaccinated risks perpetuating the current deplorable state of affairs. Second, to meet this objection on a more fundamental level, as in Bellazzi and v. Boyneburgk (2020), freedom in a virtue ethics framework is a complex concept. A full consideration of freedom can allow for virtuous behaviour even in a context of external impositions or limitations. Specifically, one must distinguish negative from positive freedom (Berlin, 2006). Negative freedom is the *freedom from* those constraints that would prevent the development and the application of virtues. Positive freedom is the *freedom to* exercise control over one’s will and to deliberately choose to perform those actions one realizes to be virtuous (Carter, 2019; Bellazzi and v. Boyneburgk, 2020). In the present context the citizens would remain able to exercise full control of their will to be directed towards obtaining the vaccination. If we assume that the agent behaves in a way that is prudent and just, then she will maintain her freedom even if the action is imposed or prescribed by law. This is because she would seek vaccination independently from its being mandatory. Accordingly, a virtuous agent can accept the mandatory vaccination, an externally imposed action, as her own free choice by making it the object of her second order will (Bellazzi and v. Boyneburgk, 2020: 7): she still has the possibility to decide to want to get vaccinated even if she is required to do so. The externally imposed action is deliberately chosen out of the individual’s virtuous character such that she herself becomes the principle of her action (cf. Aristotle and Irwin, 1999: III.1 111a22-24).

A further objection may appeal to a Principle of Least Restrictive Alternative according to which, for a given desired result in public health, the available alternative least restrictive of individual liberties ought to be preferred (Giubilini, 2018: 60). The population’s health that is part of the common good may be promoted via means less restrictive than mandatory vaccination such as persuasive information campaigns, nudges, (financial) incentives as well as mask mandates and social distancing. Concerning the latter, it is doubtful whether the continuation of mask mandates, social distancing and quarantines is actually less restrictive than mandatory vaccinations. The former have been implemented for several months now with varying force across different countries. Nevertheless, a persistent vaccination gap in many Western countries indicates vaccine uptake has come to a standstill, despite ongoing information campaigns and nudges/disincentives in the form of vaccination certificates: At the time of writing, only 68.13 per cent of the population of the USA is considered fully vaccinated, 75.42 per cent of the UK (Mathieu *et al.*, 2021). More moderate measures have so far failed to close this gap and must therefore be deemed ineffective in bringing us nearer to the end of the COVID-19 pandemic. This could motivate a virtuous ruler to support informative measures and incentives with more robust rules. It seems those still resisting vaccination cannot be convinced by information or incentives alone. In addition, Giubilini (2018: 102ff.) suggests that mandatory vaccination, while allowing for medical exemptions, promotes not only herd immunity but fairness in that everyone must bear her burden for the common good. He argues that the fair distribution of vaccines is itself a goal of public policy and that therefore vaccine mandates are not in conflict with a Principle of Least Restrictive Alternative (ibid. p.108). This notion of fairness seems to be nothing else than part of the virtue of justice, a virtue that a virtuous ruler should instil in her subjects (‘the just will be both the lawful and what is fair’, Aristotle and Irwin, 1999: V.1, 1129b1).

## Conclusion: The Virtue Ethics Case for Individual and Mandatory COVID-19 Vaccinations

In this paper, we have explored whether virtue ethics supports an individual in the choice of getting vaccinated against COVID-19 and a virtuous ruler or legislator in rendering such vaccination mandatory for her citizens. The virtues of prudence, generosity and justice indicate that an individual agent possessing these virtues will seek COVID-19 vaccination. As a result, from the perspective of virtue ethics, this choice is morally right.

Moreover, a virtuous ruler is right to impose mandatory COVID-19 vaccinations on her citizens. Considerations of prudence and bravery suggest so. By justice, she will promote a fair distribution of benefits and burdens to promote health, a precondition of happiness.

In conclusion, virtue ethical considerations warrant both the individual choice of getting vaccinated and mandatory vaccinations against COVID-19.

## Footnotes

^1^
There is some debate in the literature whether virtue ethics can determine the morally *right* action. The present paper aims to apply virtue ethical thought to vaccinations against COVID-19 instead of engaging with that more theoretical issue.

^2^
We employ the term ‘ruler’ in a generic sense to indicate whoever has the power of making vaccination against COVID-19 mandatory. This may include legislators, governments or bodies such as parliaments or other legislating institutions. Aristotle uses both the word ‘ruler’ in V.6 and ‘legislator’ in X.9 of his *Nicomachean Ethics*.

^3^
If, as we argue, the individual choice of seeking vaccination against COVID-19 is virtuous (and not just morally neutral), this fact will provide additional moral grounds for mandating vaccination against COVID-19 and rendering this policy acceptable. Showing that accepting a vaccine against COVID-19 is a virtuous choice represents further motivation and justification for a vaccination mandate. Besides, there may be virtue ethically neutral actions that a virtuous ruler is permitted to mandate. If such a ruler can mandate morally neutral actions, the same will hold *a fortiori* for virtuous actions.

^4^
While young adults have a different risk profile with regards to COVID-19, this does not imply that they have—qua moral agents—different responsibilities or should practice other virtues. The available evidence and recommendations by national health agencies indicate that the benefits of vaccinating this group outweigh the risks. Therefore, prudent young adults will seek vaccination. Prudence suggests the same course of action to both young and older adults.

^5^
While Aristotle restricts this virtue to action on the battlefield, it can be naturally extended.

^6^
One may object that young adults are at negligible risk of requiring hospitalisation and that therefore the present considerations around justice apply to them only in a diminished or qualified way. However, they may still become vectors of the virus and infect others. Moreover, justice at the societal level may require young adults to pay their due in the form of accepting vaccination. Considerations of justice indicate that everybody should contribute by getting vaccinated, individual medical condition allowing (cf. Giubilini 2018: 102ff.).

^7^
On mandatory vaccinations in a libertarian framework see Brennan (2018) and Flanigan (2014).

^8^
While the more theoretical controversy of whether and how only virtuous actions can be prescribed by the law exists, this paper does not have the space to address it in any detail.
